# Supplementary material for: For-profit growth and academic decline: a retrospective nationwide assessment of Brazilian medical schools
Source: Front Med (Lausanne). 2025 Jul 4;12:1617885. doi: 10.3389/fmed.2025.1617885 (PMC12271132; doi:10.3389/fmed.2025.1617885)
Supplement: Supplementary file 1 [file Data_Sheet_1.pdf]

Supplementary Material

1 Supplementary Figures

Supplementary Figure 1. Correlation Between ENADE Scores and Class Sizes.

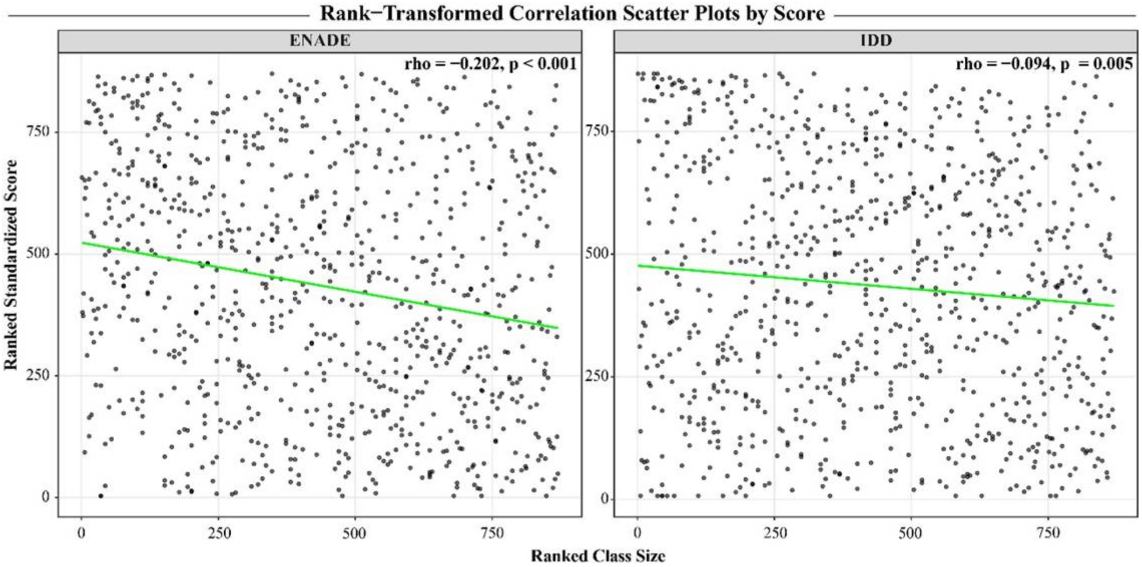

Figure Legend: Spearman Correlation scatter plots assessing the relationship between medical school class sizes and standardized scores for continuous ENADE and IDD scores. Ranked-transformed data on both standardized scores, as well as class sizes, is presented with the green line representing the direction of association. Spearman's rho and respective p-values are presented in text.

Supplementary Figure 2. Correlation Between ENADE Scores and Class Sizes According to Institutional Types.

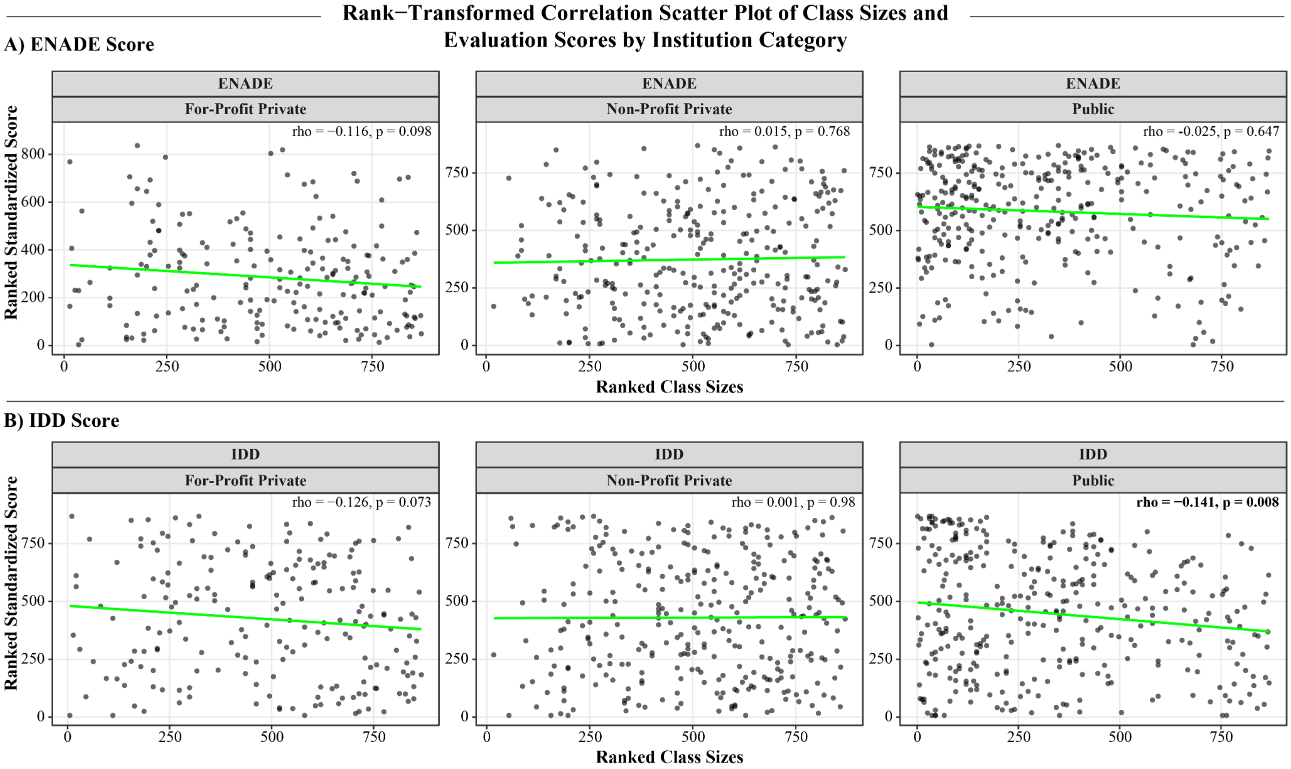

Figure Legend: Spearman Correlation scatter plots assessing the relationship between medical school class sizes and university standardized scores for continuous ENADE and IDD scores, according to their institutional types (for-profit private, non-profit private, and public institutions). Ranked-transformed data on both standardized scores, as well as class sizes, is presented with the green line representing the direction of association. Spearman's rho and respective p-values are presented in text.
